# A data-driven approach to hepatitis C forecasting using machine learning and epidemiological models

**DOI:** 10.1371/journal.pone.0357173

**Published:** 2026-08-31

**Authors:** Abdul Mannan, Jamshaid Ul Rahman, Ebraheem Alzahrani, Osman Abubakar Fiidow

**Affiliations:** 1 Abdus Salam School of Mathematical Sciences, Government College University, Lahore, Pakistan; 2 Department of Mathematics, Faculty of Science, King Abdulaziz University, Jeddah, Saudi Arabia; 3 Department of Public Health Faculty of Health Science Salaam University, Mogadishu, Somalia; Gazipur Agricultural University, BANGLADESH

## Abstract

The hepatitis C virus is a significant global health concern and a major cause of chronic liver disease. Therefore, developing a mathematical model is essential for understanding, controlling and managing its transmission dynamics. In this paper, we developed a mathematical model and simulated the dynamics of transmission of the hepatitis C virus, considering alcohol use, a key factor contributing to increased damage to the liver of infected individuals. Computational investigation of nonlinear biological models by traditional numerical solvers is challenging due to their nonlinearity and inherent complexity. Furthermore, we proposed a deep learning-based technique to solve the nonlinear differential equations governing the model for transmission of the hepatitis C virus in six compartments. The proposed deep learning-based model is compared with other established methods like the Runge-Kutta method and Livermore solver for ordinary differential equations algorithm to validate its efficacy and accuracy in predicting hepatitis C dynamics. The basic reproduction number, *R*_0_, is calculated and stability is analyzed at both the disease-free and endemic equilibrium points. Sensitivity analysis is performed to determine key parameters that contribute to hepatitis C transmission. The results indicate that the proposed approach is an efficient and robust solution with better convergence and stability than existing methods. This study highlights the potential of deep learning methods in epidemiology as a promising tool for predicting and controlling infectious diseases such as hepatitis C, particularly in the presence of behavioral risk factors such as alcohol consumption.

## Introduction

Hepatitis C is a significant health burden on the health systems of the world because of the development of potentially life-threatening conditions if the disease is not treated [1]. Despite considerable advances on the fronts of diagnosis and treatment, millions of people are infected with undetected or undertreated disease [2]. The most vulnerable populations, and especially the poorest sections of society in the poorest countries, have the highest disease burden as a result of minimal availability of medical resources and public health measures [3]. Pakistan, Egypt, and China have reported especially large numbers of cases, stressing the vital necessity of increased prevention and intervention measures [4].

Initiatives towards curbing the outbreak and effects of the virus have progressed through enhanced diagnostic methods and increasingly accessible antiviral drugs. International health agencies have established lofty goals of drastically lowering infection rates and accompanying fatalities over the next decade. At the heart of these aspirations stand mass screening, timely detection, and effective treatment protocols among susceptible populations [5]. Mathematical and computational models are of fundamental importance for predicting the trends of infection and designing effective health policies, enabling a more systematic approach to combating the epidemic [6].

Chronic alcohol use accelerates the development of liver injury in hepatitis C-infected individuals towards the development of severe liver disease [7,8]. Evidence shows that alcohol abusers have a higher likelihood of developing advanced liver fibrosis and cirrhosis than non-users [9]. Alcohol use and infection by HCV coexist for most cases, especially among those who have pre-existing liver conditions [10]. Alcohol use and disease management are made more complex by this comorbidity and present a formidable barrier towards the delay of hepatic damage progression, especially among populations where alcohol use is common [11].

Additionally, alcohol consumption seems to impair the success of antiviral therapies for the treatment of hepatitis C infection [12]. While the precise relationship is intricate, evidence indicates that heavy drinkers have weaker responses towards interferon-based regimens. Long-term alcohol abstinence, on the other hand, appears to be linked with enhanced therapeutic outcomes [13]. Some studies point towards the fact that alcohol abstinence over time augments the likelihood of a sustained virologic response (SVR), emphasizing the necessity of integrated treatment regimens that cover substance abuse as well as viral eradication [14].

Our model assumptions are reinforced by biological evidence indicating that high alcohol consumption exacerbates liver damage in individuals with HCV, leading to a higher likelihood of progression to chronic disease [15]. The model itself is a modification of existing compartmental models, specifically adapted to incorporate the effects of alcohol consumption on HCV transmission dynamics. The present research was carried out to determine the impact of low- and high-quantity alcohol consumption on patients infected with HCV. The mathematical model SFBHCR consists of six coupled nonlinear differential equations. Finding simulations of this type of ODE system is challenging. Artificial neural networks (ANNs), which are computationally effective approaches, can be used to determine the simulations of these differential equations.

According to the universal approximation theorem, every continuous function can be accurately approximated to an arbitrary level by a neural network having at least one hidden layer with a finite number of neurons and a nonlinear activation function [16]. A feedback mechanism, which is also called a backpropagation algorithm [17,18], is utilised by neural networks. Based on the discrepancy between the network’s actual and expected output, each unit’s weights are modified. These methods have continuous and differentiable solutions, show good interpolation features, and use less memory [19,20]. The necessity to retain just the weights of the neural network makes it less memory-intensive.

Once trained, the neural network provides a closed-form solution, allowing the answer to be recovered at any point within the solution domain. Having an easy-to-use application that helps researchers quickly set up and solve issues would be tremendously helpful, especially given the interest in developing neural networks to solve differential equations. Universal function approximators are multilayer perceptrons, the most basic kind of artificial neural networks (ANNs) [21]. This suggests that using ANNs to simulate the differential equations would be feasible. Previous studies have demonstrated that ANNs may simulate ordinary differential equations (ODEs) under certain initial/boundary conditions [22]. The inverse partial differential equations are simulated using ANNs [23–25], and partial differential equations in large dimensions are simulated [26,27]. Enforcing continuous symmetries in neural networks with knowledge of physics to solve forward and inverse PDE issues [28].

The neural network method is used for the parameter estimation of fractional discrete-time unified systems [29]. An artificial neural network framework for estimating the ideal value of the stabilisation parameter has been suggested [30]. Explain and illustrate the perceptron convergence technique for complex multivalued neural networks (BMVNNs), as well as several other important discoveries in the science of neural networks based on complex algebra [31]. Multi-layer neural networks are used to learn fractional difference equations through data-driven techniques [32]. Recently, researchers have addressed the Ebola virus disease model with a non-linear incidence rate using a Morlet wavelet neural network [33–35] and AI framework for opportunistic screening, staging, and progression risk stratification of steatotic liver disease [36]. Deep learning-based techniques have been used for the computational investigation and modelling of Marburg virus epidemics for health care [37] and the Van der Pol-Mathieu-Duffing oscillator model [38]. ANNs are utilised in the simulation and approximation of the biophysical model [39]. A deep neural network (DNN) is a special type of artificial neural network having two or more hidden layers.

**Fig 1:**
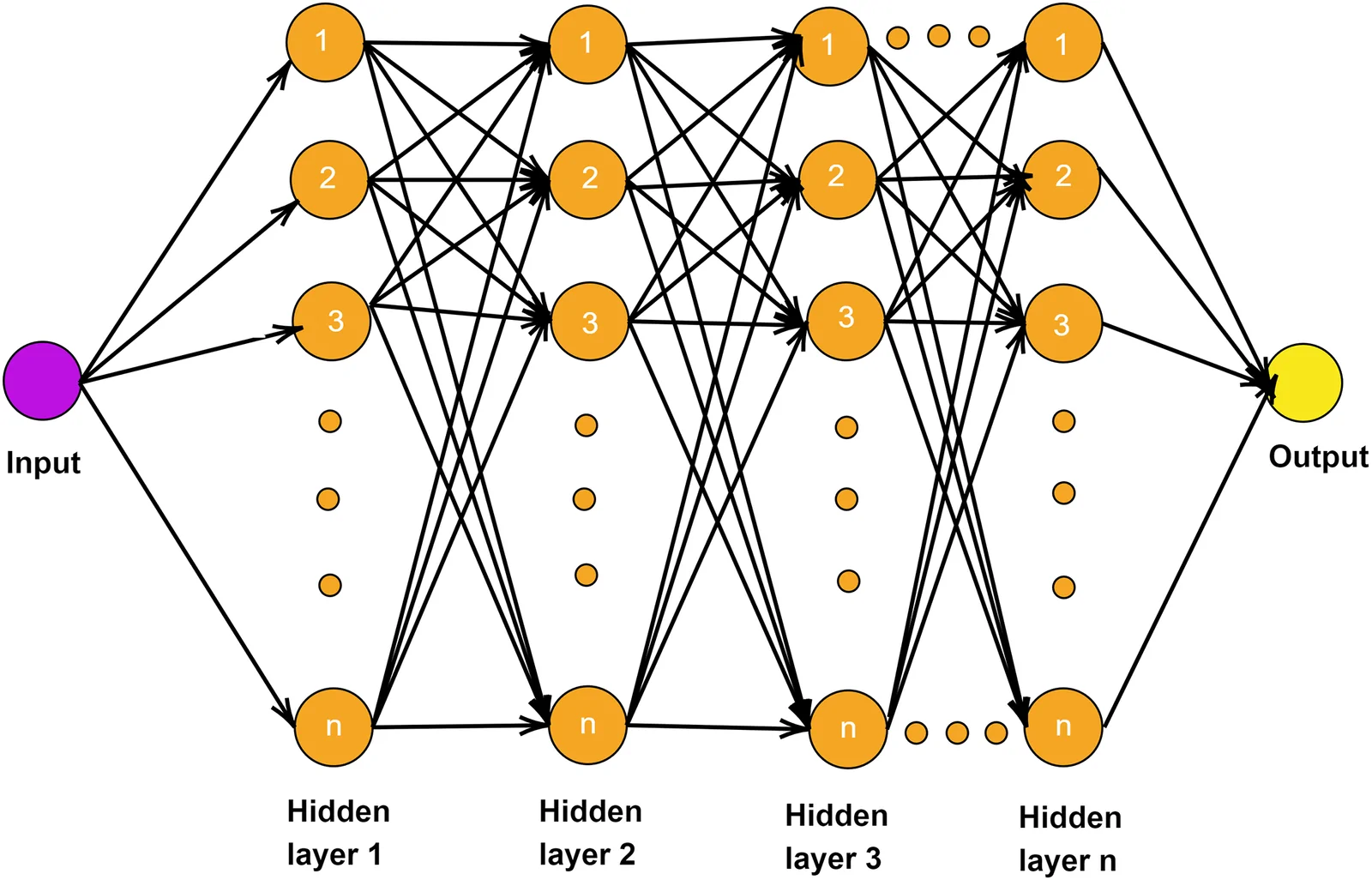
The schematic diagram of DNNs architecture.

Applications for deep neural networks in science and engineering are numerous and include voice recognition, picture processing, handwriting analysis, signature verification, and stock market forecasting, reflecting their role as interpretable deep learning models in modern computational systems [40]. The discretization of the domain is a necessary step in traditional approaches like the finite volume method (FVM), the Adams-Bashforth-Moulton approach [41], the finite element method (FEM), the finite difference method (FDM) [42], and the Runge-Kutta forward-backward sweep approach [43]. This is a challenging task that becomes more difficult with higher dimensions. Even though the DNNs approach does not require the discretization of the domain or use a mesh, it helps to mitigate some of the shortcomings of more conventional numerical methods. Fig 1 illustrates the architecture of the DNN, which consists of *n* hidden layers, each of which has *n* collaborating neurons. There is a single input layer, two or more hidden layers, and a single output layer in this network.

This paper investigates the transmission dynamics of the hepatitis C virus (HCV) using an oscillatory deep neural network-based approach, employing the SFBHCR model. The paper is structured as follows: Section outlines the mathematical modelling of a nonlinear coupled mathematical model for HCV transmission in the context of alcohol consumption. Section discusses the virus-free equilibrium point, the endemic equilibrium point, and the basic reproduction number *R*_0_. Stability analyses at both equilibrium points are examined in Section. Section consists of sensitivity analysis, which is conducted to identify the most influential parameters in the model. In Section, the proposed methodology is explained, including the architecture of oscillatory deep neural networks (ODNNs) and an algorithm detailing how ODNNs simulate the HCV transmission model. Section presents a numerical analysis of the HCV transmission model, comparing the outputs of the model using ODNNs against the RK4 method and the LSODA algorithm. Finally, Section summarises the conclusions drawn from the study and future direction.

## Model formulation

**Fig 2:**
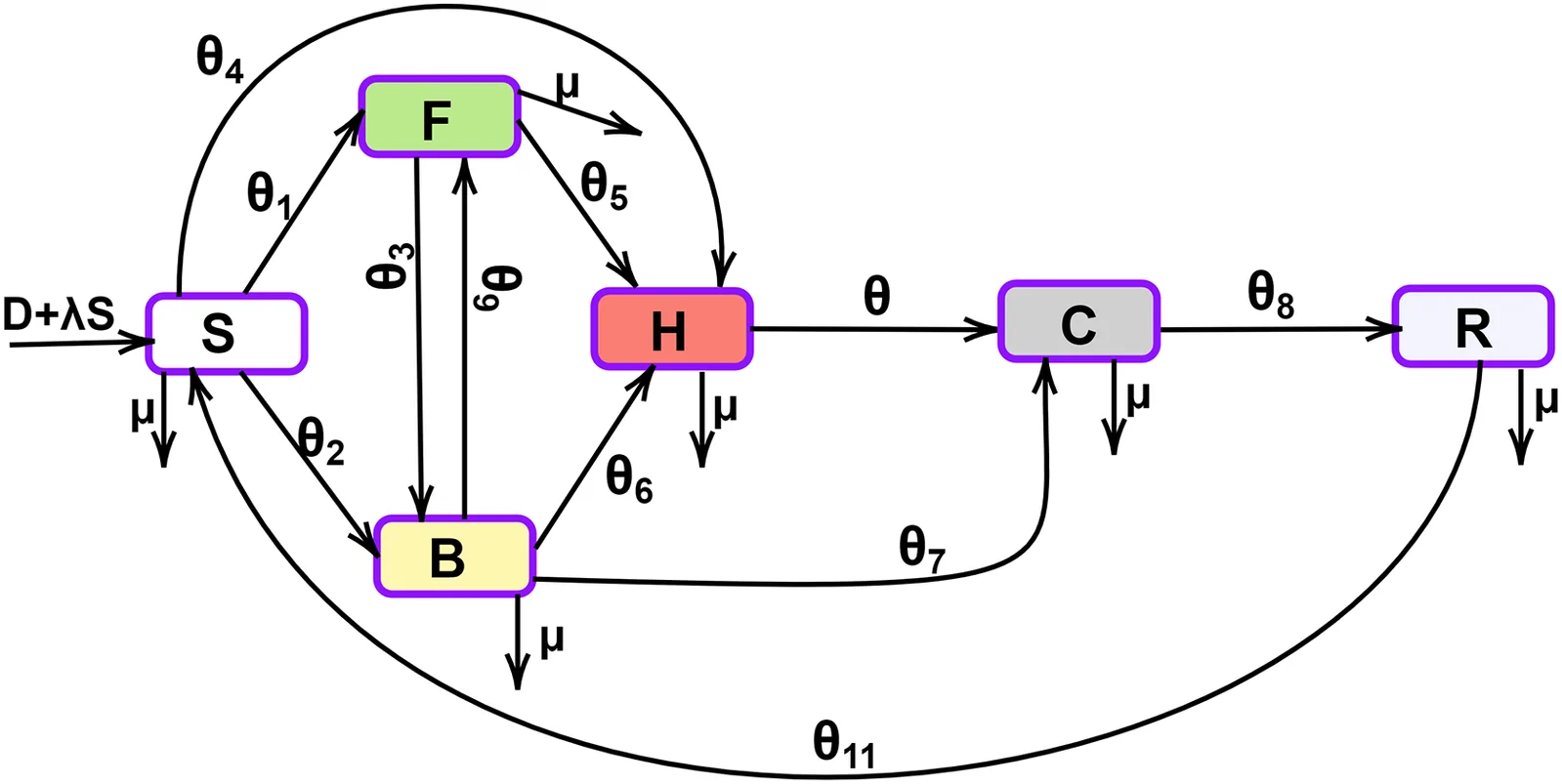
Flow diagram of the SFBHCR model illustrating HCV transmission dynamics and the influence of alcohol consumption patterns.

A mathematical model was constructed to describe the recovery dynamics of HCV-infected individuals as a function of alcohol intake. The model classifies individuals according to the alcohol they drink: those who take small quantities of alcohol (less than one shot) and those who take large quantities (more than one shot). As illustrated by Fig 2, the model follows the dynamics of the movement of the individuals between various compartments.

New susceptible individuals are added into the model at a rate of λS. Individuals who take alcohol at the lower level move into the *F* compartment at a transition rate of $\theta_{1}$, and those who are heavy drinkers move into the *B* compartment at the rate of $\theta_{2}$. The transitions from *S* to *F* at rate $\theta_{1}$ and from *S* to *B* at rate $\theta_{2}$ represent behavioral shifts influenced by social and environmental factors rather than direct infection.

Alcohol consumption, driven by peer pressure or personal choices, increases engagement in risky behaviors such as needle sharing or unprotected contact, which heightens HCV transmission risk. Biologically, excessive alcohol weakens the immune system, making individuals more susceptible to infections. These transitions reflect how individuals gradually move into higher risk categories, even without initial exposure to the virus. Thus, the model captures an indirect but significant pathway that contributes to the spread of HCV. The transitions from *F* and *B* to *H* at rates $\theta_{5}$ and $\theta_{6}$ are biologically justified by the strong correlation between alcohol use and high-risk behaviors leading to HCV transmission.

Studies show that alcohol consumption significantly increases the likelihood of engaging in unsafe practices, such as needle sharing, unprotected contact, or other risky behaviors that directly expose individuals to HCV. In addition, alcohol impairs judgment and decision-making, further increasing the probability of infection. These transitions explain the epidemiological reality that individuals in *F* and *B* have a higher probability of contracting HCV due to behavioral risks rather than direct force of infection alone. Thus, $\theta_{5}$ and $\theta_{6}$ reflect the real-world pathways through which alcohol consumption indirectly but substantially contributes to the acquisition of HCV.

Susceptible (*S*) move into the hyperacute compartment (*H*) at the rate $\theta_{4}$. The small alcohol drinkers (*F*) have the option of transiting into the large alcohol drinkers (*B*) at the rate $\theta_{3}$, while the large alcohol drinkers (*B*) have the ability of decreasing the quantity of alcohol they intake and coming back into the category of small alcohol drinkers (*F*) at the rate $\theta_{9}$. The hyperacute compartment (*H*) progresses to the chronic compartment (*C*) at the rate θ. The chronic compartment (*C*) recovers at the rate $\theta_{8}$. Recovered compartment (*R*) individuals may return to susceptibility at rate $\theta_{11}$. *D* and μ refer to the “new recruitment rate” and the “death rate,” respectively. The model of the system represented by Fig 2 is as follows:


$$\begin{array}{cc} \frac{dS}{dt} & =D+\lambda S+\theta_{11}R-\theta_{4}S-\theta_{1}S-\theta_{2}S-\mu S,\\ \frac{dF}{dt} & =\theta_{1}S-\theta_{5}F-\theta_{3}F+\theta_{9}B-\mu F,\\ \frac{dB}{dt} & =\theta_{2}S+\theta_{3}F-\theta_{9}B-\theta_{7}B-\theta_{6}B-\mu B,\\ \frac{dH}{dt} & =\theta_{5}F+\theta_{6}B+\theta_{4}S-\theta H-\mu H,\\ \frac{dC}{dt} & =\theta H+\theta_{7}B-\theta_{8}C-\mu C,\\ \frac{dR}{dt} & =\theta_{8}C-\theta_{11}R-\mu R, \end{array}$$ (1)

where $\lambda =\frac{\alpha (\alpha_{1}F+\alpha_{2}B+\alpha_{3}H+\alpha_{4}C)}{N}$ with *S* > 0, F≥0, B≥0, C≥0, H≥0, R≥0 and *S* + *F* + *H* + *B* + *C* + *R* = *N*. λ represents the recruitment rate of susceptible individuals into the population, which biologically accounts for new individuals entering the system through birth or immigration. It determines the influx of susceptible who may later become infected, impacting HCV transmission dynamics. Tables 1 and 2 explain the parameters of the SFBHCR model. By adding the above system of Eq 1 and we get,


$$\begin{array}{c} \frac{d}{dt}(S+F+B+H+C+R)=D-\mu (S+F+B+H+C+R)\geq 0 \end{array}$$ (2)

**Table 1:** Model variables and description.

Notation	Description	Units
*S*(*t*)	Number of susceptible individuals at any time *t*	Individuals
*F*(*t*)	Number of individuals consuming alcohol in small quantities at any time *t*	Individuals
*B*(*t*)	Number of individuals consuming alcohol in large quantities at any time *t*	Individuals
*H*(*t*)	Number of hyperacutely infected individuals at any time *t*	Individuals
*C*(*t*)	Number of chronically infected individuals at any time *t*	Individuals
*R*(*t*)	Number of recovered individuals at any time *t*	Individuals

**Table 2:** Model parameters.

Notation	Description	Value	Units	Source
*D*	Recruitment rate	1.2	$\frac{\text{Individuals}}{\text{day}}$	[44]
θ	Hyperacute compartment progresses to chronic compartment at this rate	0.5	*day* ^-1^	[12]
$\theta_{1}$	Susceptibles move to the small alcohol consumption compartment at this rate	0.6	*day* ^-1^	[12]
$\theta_{2}$	Susceptibles move to the large alcohol consumption compartment at this rate	0.2	*day* ^-1^	[12]
$\theta_{3}$	Transition from small to large alcohol consumption compartment	0.5	*day* ^-1^	assumed
$\theta_{4}$	Susceptibles become hyperacute infected with HCV at this rate	0.35	*day* ^-1^	[13]
$\theta_{5}$	Small alcohol consumers become hyperacute infected with HCV at this rate	0.1	*day* ^-1^	[13]
$\theta_{6}$	Large alcohol consumers become hyperacute infected with HCV at this rate	0.5	*day* ^-1^	[13]
$\theta_{7}$	Large alcohol consumers become chronically infected with HCV at this rate	0.3	*day* ^-1^	assumed
$\theta_{8}$	Chronic compartment progresses to recovered compartment at this rate	0.6	*day* ^-1^	assumed
$\theta_{9}$	Transition from large to small alcohol consumption compartment	0.3	*day* ^-1^	[11]
$\theta_{11}$	Recovered compartment returns to susceptible at this rate	0.1	*day* ^-1^	[11]
μ	Natural death rate	0.01	*day* ^-1^	[44]
λ	Effective rate of contact	0.40	*day* ^-1^	assumed

Eq 2 gives Eq 3,


$$\begin{array}{c} \lim_{t\to \infty }sup(S+F+B+H+C+R)\leq \frac{D}{\mu }. \end{array}$$ (3)

Therefore, Eq 4 shows the viable area for the above systme of Eq 1 is,


$$\begin{array}{c} A=\{(S,F,B,H,C,R)/S+F+B+H+C+R\leq \frac{D}{\mu },S>0,F\geq 0,B\geq 0,C\geq 0,H\geq 0,R\geq 0\}. \end{array}$$ (4)

### Dimensional analysis of model

For verification of the accuracy and uniformity of the presented model, we now do a dimensional system of Eq 1 analysis. Analysis ensures that all the terms in every differential equation are dimensionally homogeneous, required for the biological interpretability of the model.

The state variables all represent the number of individuals. Accordingly, the dimensions of the state variables are


[S]=[F]=[B]=[H]=[C]=[R]=Individuals.

The dimensions of the model’s parameters are given as:


$$[D]=\text{Individuals}\cdot {day}^{-1},\;[\theta_{i}]=[\lambda ]=[\mu ]={day}^{-1}.$$

First of all, consider the susceptible compartment:


$$\begin{array}{c} \frac{dS}{dt}=D+\lambda S+\theta_{11}R-\theta_{4}S-\theta_{1}S-\theta_{2}S-\mu S. \end{array}$$

On the left-hand side, ($[\frac{dS}{dt}]=\text{Individuals}\cdot {day}^{-1}$). All of the terms on the right-hand side also have the same dimension $\left(\text{Individuals}\cdot {day}^{-1}\right)$.

Recalling the test on the remaining compartments (*F*, *B*, *H*, *C*, *R*) establishes that all of the terms in the system are dimensionally consistent. Thus, system of Eq 1 is dimensionally homogeneous, both sides of every equation in ($\text{Individuals}\cdot {day}^{-1}$). This attests to the mathematical stability and biological importance of the model.

### Equilibrium analysis

By using stability analysis, which is based on the system of Eq. 1, the disease-free equilibrium point can be found. System of Eq 1 must all equal zero to determine the equilibrium point, which means:


$$\begin{array}{cc} D+\lambda S+\theta_{11}R-\theta_{4}S-\theta_{1}S-\theta_{2}S-\mu S & =0,\\ \theta_{1}S-\theta_{5}F-\theta_{3}F+\theta_{9}B-\mu F & =0,\\ \theta_{2}S+\theta_{3}F-\theta_{9}B-\theta_{7}B-\theta_{6}B-\mu B & =0,\\ \theta_{5}F+\theta_{6}B+\theta_{4}S-\theta H-\mu H & =0,\\ \theta H+\theta_{7}B-\theta_{8}C-\mu C & =0,\\ \theta_{8}C-\theta_{11}R-\mu R & =0. \end{array}$$ (5)

Now, we determined disease free equilibrium and endemic equilibrium points

### Virus free equilibrium point

The equilibrium points of a Virus free state are those points where disease does not spread, which are *F* = 0, *B* = 0, *H* = 0 and *C* = 0. After solving the system of Eq 5, we determine the Virus free equilibrium point that is $P_{1}=(S_{1},F_{1},B_{1},H_{1},C_{1},R_{1})=(\frac{D}{\mu },0,0,0,0,0)$.

### Endemic equilibrium point

After solving the system of Eq 5, we determine the endemic equilibrium point that is given by Eq 6,


$$\begin{array}{c} P_{2}=(S_{2},F_{2},B_{2},H_{2},C_{2},R_{2}), \end{array}$$ (6)

where


$$\begin{array}{cc} S_{2} & =\frac{1}{2(k_{2}[\alpha_{1}m_{4}+\alpha_{2}m_{3})k_{1}+\alpha_{3}m_{1}k_{2}+\alpha_{4}m_{2}]k_{3}\alpha }*Z,\\ F_{2} & =\frac{m_{1}S_{2}}{k},\\ B_{2} & =\frac{m_{3}S_{2}}{k},\\ H_{2} & =\frac{m_{1}S_{2}}{kk_{1}},\\ C_{2} & =\frac{m_{2}S_{2}}{kk_{1}k_{2}},\\ R_{2} & =\frac{m_{1}S_{2}}{kk_{1}k_{2}k_{3}}, \end{array}$$

and

Z = {$\begin{array}{c} \begin{array}{c} -4N(k_{1}(\frac{-1}{4}k_{1}k_{2}k(\theta_{4}+\theta_{1}+\theta_{2}+\lambda {)}^{2}N+k(((\alpha_{1}m_{4}+\alpha_{2}m_{3})k_{1}+\alpha_{3}m_{1})k_{2}+\alpha_{4}m_{2})D\alpha )kk_{2}k_{3}^{2}+\frac{1}{2}m_{5}k_{1}k_{2}N\theta_{11}k(\theta_{4}+\theta_{1}+\theta_{2}+\mu )k_{3} \end{array} \end{array}$$\begin{array}{c} \begin{array}{c} -\frac{1}{4}N\theta_{11}^{2}m_{5}^{2}){)}^{\frac{1}{2}}+(\theta_{4}+\theta_{1}+\theta_{2}+\mu )kNk_{1}k_{2}k_{3}-\theta_{11}m_{5}N\} \end{array} \end{array}$,

$Y_{1}=\theta_{1}+\theta_{2}+\theta_{4}+\mu$, $Y_{2}=\theta_{5}+\theta_{3}+\mu$, $Y_{3}=\theta_{9}+\theta_{6}+\theta_{7}+\mu$, $Y_{4}=\theta +\mu$, $Y_{5}=\theta_{8}+\mu$, $Y_{6}=\theta_{11}+\mu$,

$k=\theta_{3}\theta_{9}-Y_{2}Y_{3}$, $k_{1}=Y_{4}$, $k_{2}=Y_{5}$, $k_{3}=Y_{6}$, $m_{1}=\theta_{4}\theta_{3}\theta_{9}-\theta_{4}Y_{2}Y_{3}-\theta_{3}\theta_{1}\theta_{6}-\theta_{1}\theta_{5}Y_{3}-\theta_{2}\theta_{6}Y_{2}$

$m_{2}=\theta \theta_{3}\theta_{9}\theta_{4}-\theta \theta_{4}Y_{2}Y_{3}-\theta_{7}\theta_{3}\theta_{1}Y_{4}-\theta_{7}\theta_{2}Y_{2}Y_{4}-\theta \theta_{3}\theta_{1}\theta_{6}-\theta \theta_{9}\theta_{2}\theta_{5}-\theta \theta_{5}\theta_{1}Y_{3}-\theta \theta_{2}\theta_{6}Y_{2}$,

$m_{3}=\theta_{3}\theta_{1}+\theta_{2}Y_{2}$, $m_{4}=\theta_{9}\theta_{2}+\theta_{1}Y_{3}$,

$m_{5}=\theta_{8}(\theta \theta_{3}\theta_{9}\theta_{4}-\theta \theta_{4}Y_{2}Y_{3}-\theta_{7}\theta_{3}\theta_{1}Y_{4}-\theta_{7}\theta_{2}Y_{2}Y_{4}-\theta \theta_{6}\theta_{3}\theta_{1}-\theta \theta_{9}\theta_{5}\theta_{2}-\theta \theta_{5}\theta_{1}Y_{3}-\theta \theta_{6}\theta_{2}Y_{2})$.

The basic reproduction number, *R*_0_, is a key epidemiological threshold parameter used to characterize the dynamics of the endemic model. It represents the average number of secondary infections produced by a single infectious individual in a completely susceptible population. The value of *R*_0_ governs the qualitative behavior of disease transmission. In particular, if *R*_0_ < 1, the disease will eventually die out, whereas if *R*_0_ > 1, the disease persists and becomes endemic in the population. Consequently, public health interventions such as vaccination, treatment strategies, and quarantine measures are often designed and evaluated based on the magnitude of *R*_0_. Theorem 1 proves the disease-free equilibrium’s stability by showing eradication of the infection as a result of keeping *R*_0_ < 1. Through the next generation matrix method [45,46], *R*_0_ equals the spectral radius of the matrix *gq*^-1^, where *g* and *q* are the Jacobians of *G* and *Q*, respectively. Let the state of the system be defined as *Y* = (*S*, *F*, *B*, *H*, *C*, *R*).

Therefore,


$$\begin{array}{c} \frac{dY}{dt}=G(Y)-Q(Y), \end{array}$$

where Q(x) indicates how people move among compartments and G(X) indicates the rate at which new cases of infection are produced. This gives us


$$\begin{array}{c} \begin{array}{cc} G=[\begin{array}{c} \theta_{1}S\\ \theta_{2}S\\ \theta_{4}S\\ 0\\ 0\\ D+\lambda S \end{array}], & Q=[\begin{array}{c} (\theta_{5}+\theta_{3}+\mu )F-\theta_{9}B\\ -\theta_{3}F+(\theta_{9}+\theta_{7}+\theta_{6}+\mu )B\\ -\theta_{5}F-\theta_{6}B+(\theta +\mu )H\\ -\theta H-\theta_{7}B+(\theta_{8}+\mu )C\\ -\theta_{8}C+(\theta_{11}+\mu )R\\ -\theta_{11}R+(\theta_{4}+\theta_{1}+\theta_{2}+\mu )S \end{array}] \end{array}. \end{array}$$

At virus free equilibrium point $P_{1}=(\frac{D}{\mu },0,0,0,0,0)$, the derivatives of *G* and *Q* are now calculated, obtaining matrices *g* and *q* which are:


$$\begin{array}{c} g=[\begin{array}{cccccc} 0 & 0 & 0 & 0 & 0 & \theta_{1}\\ 0 & 0 & 0 & 0 & 0 & \theta_{2}\\ 0 & 0 & 0 & 0 & 0 & \theta_{4}\\ 0 & 0 & 0 & 0 & 0 & 0\\ 0 & 0 & 0 & 0 & 0 & 0\\ \frac{D\alpha \alpha_{1}}{\mu N} & \frac{D\alpha \alpha_{2}}{\mu N} & \frac{D\alpha \alpha_{3}}{\mu N} & \frac{D\alpha \alpha_{4}}{\mu N} & 0 & 0 \end{array}], \end{array}$$

and


$$\begin{array}{c} q=[\begin{array}{cccccc} \theta_{5}+\theta_{3}+\mu & -\theta_{9} & 0 & 0 & 0 & 0\\ -\theta_{3} & \theta_{9}+\theta_{7}+\theta_{6}+\mu & 0 & 0 & 0 & 0\\ -\theta_{5} & \theta_{6} & \theta +\mu & 0 & 0 & 0\\ 0 & -\theta_{7} & -\theta & \theta_{8}+\mu & 0 & 0\\ 0 & 0 & 0 & \theta_{8} & \theta_{11}+\mu & 0\\ \theta_{4}+\theta_{1}+\theta_{2}+\mu & 0 & 0 & 0 & 0 & -\theta_{11} \end{array}]. \end{array}$$

Thus, the required basic reproduction number is given by Eq 7


$$\begin{array}{c} R_{0}=\frac{-(DY_{6}\alpha \alpha_{4}(U_{1}\theta_{1}+U_{2}\theta_{2}-U_{3}\theta_{4})\theta +(U_{4}\theta_{3}+U_{5}Y_{3})\theta_{1}+(U_{4}Y_{2}+U_{5}\theta_{9})\theta_{2}-\theta_{4}\alpha_{3}U_{3}Y_{5}+\alpha_{4}\theta_{7}Y_{4}U_{6})}{\left((\theta_{11}\theta_{8}(U_{1}\theta_{1}+U_{2}\theta_{2}-U_{3}\theta_{4})\theta +Y_{4}(Y_{6}Y_{1}Y_{5}U_{3}+\theta_{7}\theta_{8}\theta_{11}U_{6}))\mu N\right)} \end{array}$$ (7)

where,

$U_{1}=\theta_{3}\theta_{6}+\theta_{5}Y_{3}$, $U_{2}=\theta_{9}\theta_{5}+\theta_{6}Y_{2}$, $U_{3}=\theta_{3}\theta_{9}-Y_{2}Y_{3}$, $U_{4}=\alpha_{2}Y_{4}+\alpha_{3}\theta_{6}$, $U_{5}=\alpha_{1}Y_{4}+\alpha_{3}\theta_{5}$, $U_{6}=\theta_{3}\theta_{1}+\theta_{2}Y_{2}$ and $Y_{1},Y_{2},Y_{3},Y_{4},Y_{5},Y_{6}$ are given in Eq 6.

### Stability analysis

This section contains the model’s stability analysis. It is now needed to determine the Jacobian matrix based on system Eq 1 to examine stability. First, we find the Jacobian matrix at the virus free equilibrium point $P_{1}=(\frac{D}{\mu },0,0,0,0,0)$ that is:


$$\begin{array}{c} J(P_{1})=[\begin{array}{cccccc} -(\theta_{4}+\theta_{1}+\theta_{2}+\mu ) & \frac{D\alpha \alpha_{1}}{\mu N} & \frac{D\alpha \alpha_{2}}{\mu N} & \frac{D\alpha \alpha_{3}}{\mu N} & \frac{D\alpha \alpha_{4}}{\mu N} & \theta_{11}\\ \theta_{1} & -(\theta_{5}+\theta_{3}+\mu ) & \theta_{9} & 0 & 0 & 0\\ \theta_{2} & \theta_{3} & -(\theta_{9}+\theta_{7}+\theta_{6}+\mu ) & 0 & 0 & 0\\ \theta_{4} & \theta_{5} & \theta_{6} & -(\theta +\mu ) & 0 & 0\\ 0 & 0 & \theta_{7} & \theta & -(\theta_{8}+\mu ) & 0\\ 0 & 0 & 0 & 0 & \theta_{8} & -(\theta_{11}+\mu ) \end{array}] \end{array}$$

The jacobian matrix *J*(*P*_1_) have the following eigenvalues:


$$\begin{array}{c} \lambda_{1}=-(\theta_{4}+\theta_{1}+\theta_{2}+\mu ), \end{array}$$ (8)

$$\begin{array}{c} \lambda_{2}=-(\theta_{5}+\theta_{3}+\mu ), \end{array}$$ (9)

$$\begin{array}{c} \lambda_{3}=-(\theta_{9}+\theta_{7}+\theta_{6}+\mu ), \end{array}$$ (10)

$$\begin{array}{c} \lambda_{4}=-(\theta +\mu ), \end{array}$$ (11)

$$\begin{array}{c} \lambda_{5}=-(\theta_{8}+\mu ), \end{array}$$ (12)

$$\begin{array}{c} \lambda_{6}=-(\theta_{11}+\mu ). \end{array}$$ (13)

It should be noted that in the proposed model, all the parameters are taken to be positive. With this, all the eigenvalues $\lambda_{1}$, $\lambda_{2}$, $\lambda_{3}$, $\lambda_{4}$, $\lambda_{5}$, and $\lambda_{6}$ of the Jacobian matrix *J*(*P*_1_) have negative real parts. It implies that *R*_0_ < 1 and the disease-free equilibrium *P*_1_ is locally asymptotically stable. Stability at the disease-free state thus ensures that there can be no outbreak or pandemic risk in the population.

Now, we find the Jacobian matrix at the endemic equilibrium point $P_{2}=(S_{2},F_{2},B_{2},H_{2},C_{2},R_{2})$that is:


$$\begin{array}{c} J(P_{2})=[\begin{array}{cccccc} \frac{\alpha (\alpha_{1}F_{2}+\alpha_{2}B_{2}+\alpha_{3}H_{2}+\alpha_{4}C_{2})}{N}-Y_{1} & \frac{D\alpha \alpha_{1}}{\mu N} & \frac{D\alpha \alpha_{2}}{\mu N} & \frac{D\alpha \alpha_{3}}{\mu N} & \frac{D\alpha \alpha_{4}}{\mu N} & \theta_{11}\\ \theta_{1} & -Y_{2} & \theta_{9} & 0 & 0 & 0\\ \theta_{2} & \theta_{3} & -Y_{3} & 0 & 0 & 0\\ \theta_{4} & \theta_{5} & \theta_{6} & -Y_{4} & 0 & 0\\ 0 & 0 & \theta_{7} & \theta & -Y_{5} & 0\\ 0 & 0 & 0 & 0 & \theta_{8} & -Y_{6} \end{array}], \end{array}$$

where $Y_{1},Y_{2},Y_{3},Y_{4},Y_{5},Y_{6}$ are given in Eq 6. Now, we determine the characteristic equation of the matrix *J*(*P*_2_). Hence, the characteristic equation is given by Eq 14


$$\begin{array}{c} b_{2}e^{2}+b_{1}e+b_{0}=0, \end{array}$$ (14)

with

$b_{0}=Dkk_{1}k_{2}N$,

$b_{1}=-(\theta_{1}kk_{1}k_{2}N+\theta_{2}kk_{1}k_{2}N+\theta_{4}kk_{1}k_{2}N-\frac{\theta_{11}m_{5}N}{k_{3}}+\mu kk_{1}k_{2}N)$,

$b_{2}=\alpha \alpha_{1}m_{4}k_{1}k_{2}+\alpha \alpha_{2}m_{3}k_{1}k_{2}+\alpha \alpha_{3}m_{1}k_{2}+\alpha \alpha_{4}m_{2}$.

Calculated the values of the coefficients by substituting parametric values given in Table 1. We get *b*_0_ = 1.0206, *b*_1_ = 0.7377, *b*_2_ = 0.006. Solving Eq 8 determined two solutions e=−1.4014 or e=−108.27 which meets Routh-Hurwitz criteria (if real roots of the characteristic equation have negative sign then the system is stable)[47]. Hence, *P*_2_ is locally asymptotically stable.

### Sensitivity analysis

The significance of each parameter in influencing the dynamics of disease transmission is analyzed through sensitivity analysis. This method helps identify key factors that have a substantial impact on the basic reproduction number *R*_0_. Specifically, the normalized forward sensitivity index of a variable with respect to a parameter quantifies the ratio of the relative change in the variable to the relative change in the parameter [48]. The normalized forward sensitivity index of *R*_0_, which is differentiable with respect to a parameter ρ, is given by:


$$\begin{array}{c} \gamma_{\rho }^{R_{0}}=\frac{\partial R_{0}}{\partial \rho }\times \frac{\rho }{R_{0}}. \end{array}$$ (15)

**Fig 3:**
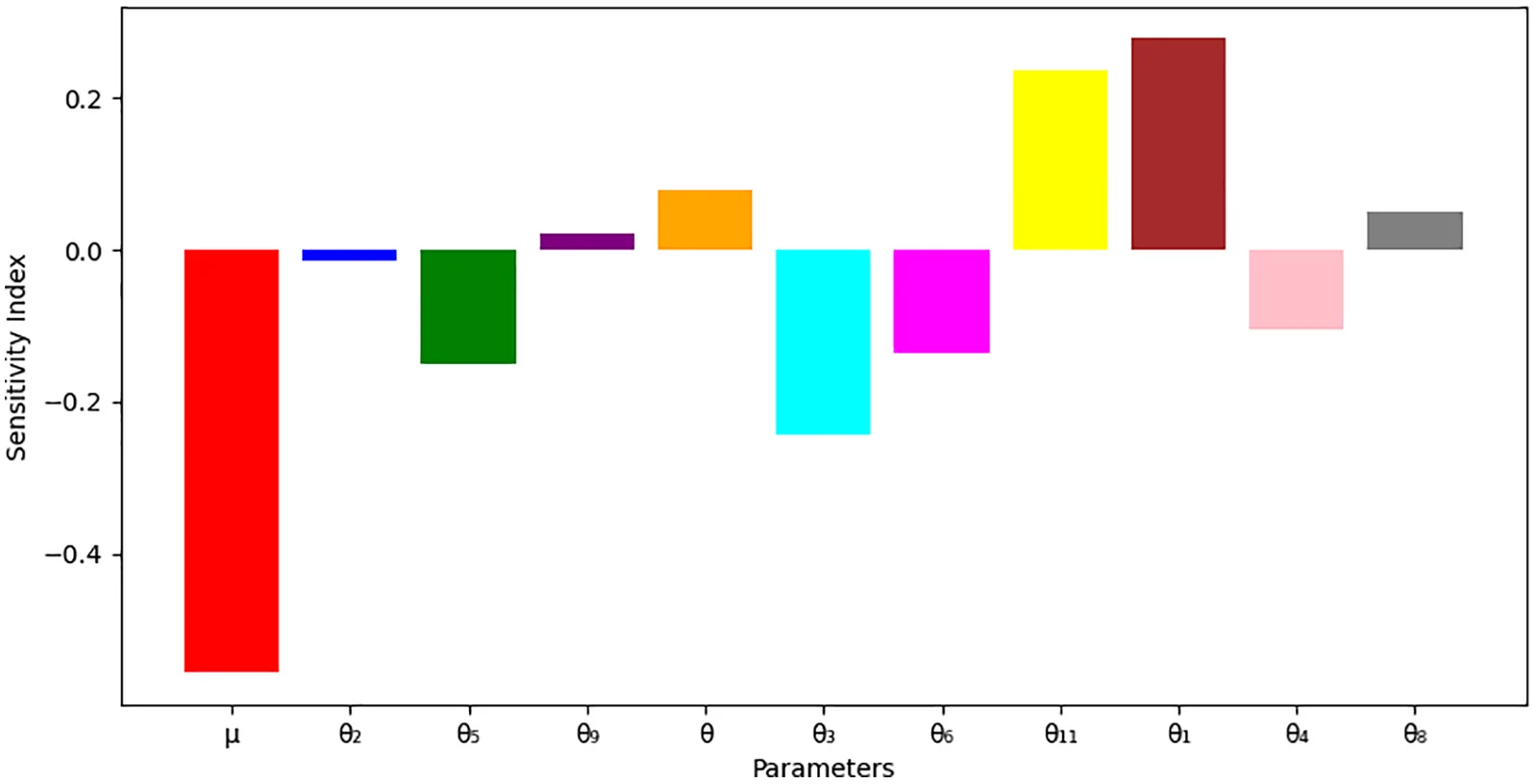
Sensitivity indices for all parameters of *R*_0_.

This index measures the relative change in the basic reproduction number *R*_0_ in response to a relative change in each parameter. It provides important insights into the sensitivity of *R*_0_ to parameter variations, facilitating the evaluation of each parameter’s impact on disease transmission dynamics. The computed sensitivity indices are given in Table 3 and graphically presented in Fig 3, demonstrating how the model outcomes respond to changes in parameters based on the baseline values given in Table 2. A positive sensitivity index indicates that an increase in the parameter leads to an increase in *R*_0_, whereas a negative value signifies that an increase in the parameter results in a decrease in *R*_0_. These sensitivity indices help determine the direction of *R*_0_ changes with respect to each parameter, aiding in the development of targeted intervention strategies for disease control. e development of targeted intervention strategies for disease control.

**Table 3:** Sensitivity indices for parameters impacting *R*_0._

Parameter	Value	Parameter	Value	Parameter	Value	Parameter	Value
μ	−0.5565	$\theta_{2}$	−0.0156	$\theta_{5}$	−0.1507	$\theta_{9}$	0.0216
θ	0.0788	$\theta_{3}$	−0.2448	$\theta_{6}$	−0.1354	$\theta_{11}$	0.2353
$\theta_{1}$	0.2780	$\theta_{4}$	−0.1060	$\theta_{8}$	0.0501		

The sensitivity analysis results in Table 3 provide important epidemiological insights into the transmission dynamics of hepatitis C virus under alcohol consumption. The parameters μ, $\theta_{3}$, $\theta_{5}$, and $\theta_{6}$ exhibit negative sensitivity indices, indicating that increasing these parameters leads to a reduction in the basic reproduction number *R*_0_, thereby contributing to disease control. In particular, the natural death rate μ shows the strongest negative influence, suggesting that factors reducing the average infectious lifespan significantly suppress disease transmission. Similarly, transition rates such as $\theta_{3}$, $\theta_{5}$, and $\theta_{6}$ reflect movement between behavioral and infected compartments, and their negative sensitivity highlights the role of reduced high-risk progression in lowering infection spread.

On the other hand, positive sensitivity indices are observed for parameters such as $\theta_{1}$, $\theta_{8}$, and $\theta_{11}$, indicating that increases in these parameters may contribute to an increase in *R*_0_. In particular, $\theta_{1}$ plays a key role in moving individuals from susceptibility into alcohol consumption related compartments, thereby indirectly increasing infection risk. The recovery related parameter $\theta_{8}$ and the relapse parameter $\theta_{11}$ also show positive contributions, suggesting that reinfection or loss of immunity may sustain transmission dynamics. Overall, these results identify key leverage points for intervention strategies, emphasizing that controlling alcohol related transitions and improving sustained recovery can significantly reduce hepatitis C transmission.

### Proposed methodology

In this section, we have examined our proposed methodology and the architecture of ODNNs, including an exploration of mean square error (MSE) employed within ODNNs to measure the disparity between actual and predicted values. Furthermore, we will explore how ODNNs simulate the SFBHCR alcohol model.

### Neural networks method

Consider *t* belonging to *D*, a subset of the real numbers ℝ, and Eq. 16 express the differential equation in its general form as:


$$G(t,v(t),v'(t),v''(t),v'''(t),...,v^{n}(t))=0.$$ (16)

In this context, *t* serves as the independent variable of the differential equation, while *v*(*t*) represents its solution. To address ordinary differential equations (ODEs) using neural networks, a trial solution is often formulated. The objective of this trial solution lies in fulfilling the prescribed initial and boundary conditions. Typically, the following general form given in Eq 17 is employed in its formulation.


$$\begin{array}{c} v_{t}(t)=Z(t)+F(t,N(t,r)). \end{array}$$ (17)

The neural network output, denoted as *N*(*t*,*r*), where *t* denotes time and *r* represents parameters (including weights and biases). Additionally, the form of the specific ordinary differential equation determines the functions *F*(*t*, *N*(*t*,*r*)) and *Z*(*t*), which fulfil the initial or boundary conditions appropriately. The neural network adjusts its weights and biases iteratively to minimize error and approximate the solution to the problem. Utilizing mean squared error, it computes and applies necessary adjustments to minimize the error effectively.

DNNs have been shown to effectively solve systems of first-order ordinary differential equations (ODEs) [49]. When dealing with a system given in Eq 18, consisting of *m* ODEs,


$$\begin{array}{c} \frac{dv_{t_{i}}(t)}{dt}=f_{i}(t,v_{1},v_{2},v_{3},...,v_{m}). \end{array}$$ (18)

With $v_{i}(0)=Z_{i}$, *i* = 1,2,3,...,*m*. Each differential equation’s trial solution is constructed by Eq 19.


$$\begin{array}{c} v_{t_{i}}(t)=Z_{i}(t)+tN_{i}(t,r_{i}),i=1,2,3,...,m. \end{array}$$ (19)

Here, the model’s effectiveness during training is evaluated by computing the average discrepancy between the expected and actual output values. Mean squared error (MSE) serves as a widely employed metric in regression problems [50]. The mathematical expression of MSE is determined by the system of ODEs specified by Eq 20.


$$\begin{array}{c} MSE=\frac{1}{n}\sum_{j=1}^{m}\sum_{i=1}^{n}(\frac{dv_{t_{j}}(t_{i})}{dt}-f_{j}(t_{i},v_{t_{1}},u_{t_{2}},v_{t_{3}},...,v_{t_{m}}){)}^{2}. \end{array}$$ (20)

### ODNNs framework

**Fig 4:**
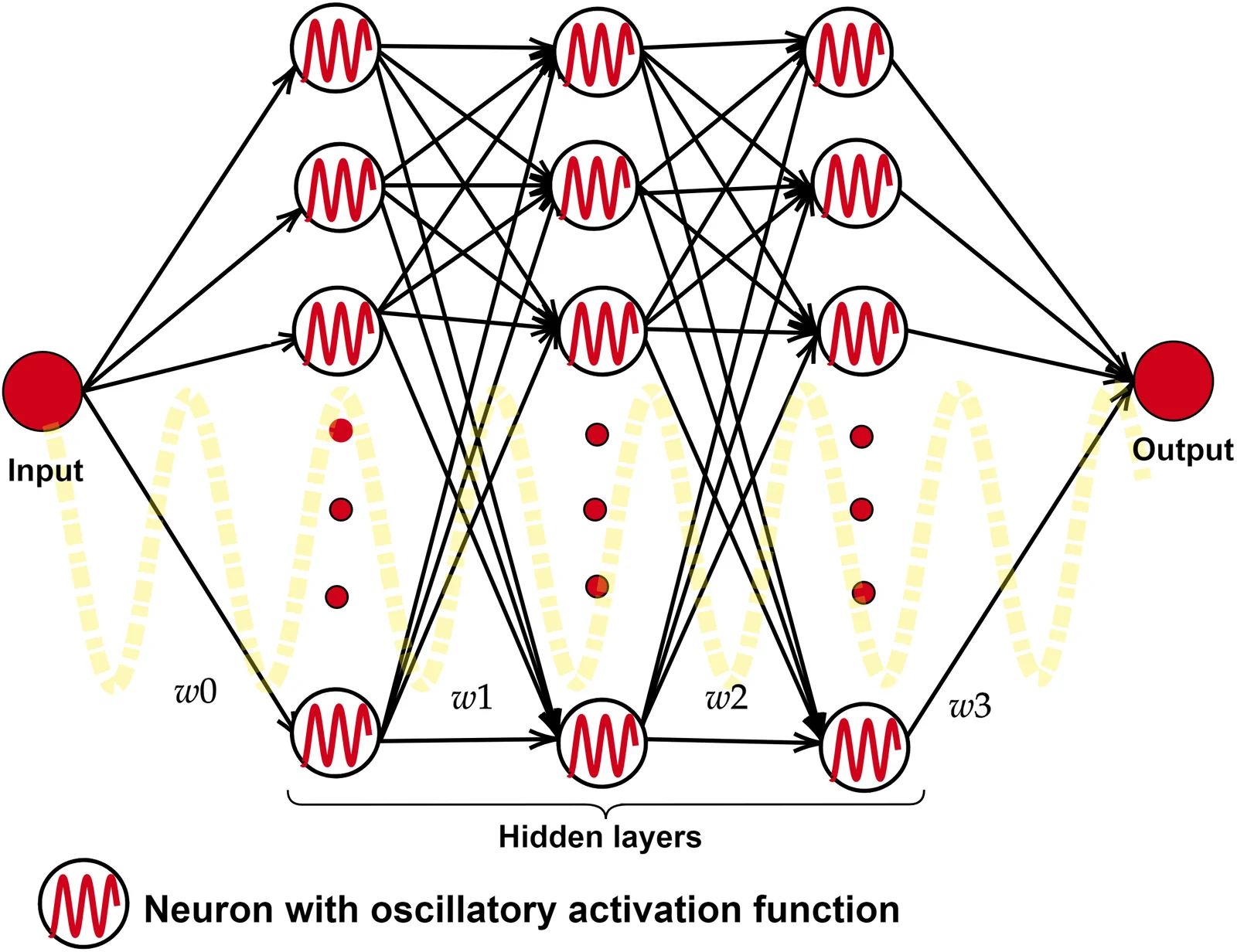
The schematic diagram of the proposed oscillatory deep neural network architecture.

Using ODNNs to approximate the SFBHCR model for HCV transmission under boozing. The design, as illustrated in Fig 4, comprises building an input layer with initial conditions and model parameters, then three hidden layers, each with 32 neurons, and an output layer that predicts how *S*, *F*, *B*, *H*, *C*, and *R* compartments will change over time. In the hidden layers, we use the sine activation function [50]. In order to improve the network’s ability to capture complex nonlinear dynamics in the SFBHCR model, the sine activation function is employed to enhance transformations between neurons. The choice of oscillatory activation is motivated by the oscillatory nature of biological neural activity, where neuronal firing often exhibits rhythmic behavior. In contrast, standard activation functions such as ReLU and Sigmoid are non-oscillatory and may struggle to represent periodic or wave-like dynamics commonly observed in epidemiological systems [51].

When the underlying dynamics are oscillatory or periodic, the sine function provides a more suitable representation due to its inherent periodicity. Unlike ReLU and Sigmoid, it naturally captures cyclic behavior, leading to improved approximation of nonlinear epidemic processes. Its smoothness, boundedness, and periodic structure contribute to stable and accurate learning in continuous dynamical models. This justifies the use of ODNNs in this study, particularly for epidemiological forecasting problems where conventional machine learning approaches are limited in capturing oscillatory behavior. Additionally, two essential components are integrated into the formulation of the loss function. The boundary/initial condition loss penalizes differences between specified initial conditions and predicted conditions, and the ordinary differential equation Residual loss quantifies deviations between predicted and SFBHCR-derived derivatives for *S*, *F*, *B*, *H*, *C*, and *R* compartments.

The ODNNs train iteratively via data generation, weight initialization, optimization [52] using algorithms such as Adam (adaptive moment estimation), and parameter adjustments through backpropagation [53]. Accuracy and robustness in predicting HCV transmission dynamics are ensured by hyperparameter fine-tuning along with validation using SFBHCR model solutions. For the SFBHCR model, we can build the trial solution as Eq 21.


$$\begin{array}{cc} v_{t_{s}}(t) & =S_{0}+tN_{s}(t,p_{s}),\\ v_{t_{f}}(t) & =F_{0}+tN_{f}(t,p_{f}),\\ v_{t_{b}}(t) & =B_{0}+tN_{b}(t,p_{b}),\\ v_{t_{h}}(t) & =H_{0}+tN_{h}(t,p_{h}),\\ v_{t_{c}}(t) & =C_{0}+tN_{c}(t,p_{c}),\\ v_{t_{r}}(t) & =R_{0}+tN_{r}(t,p_{r}), \end{array}$$ (21)

with *S*(0)=*S*_0_, *F*(0)=*F*_0_, *B*(0)=*B*_0_, *H*(0)=*H*_0_, *C*(0)=*C*_0_, *R*(0)=*R*_0_.

### Algorithm

How the SFBHCR model is simulated using ODNNs. The parameters of ODNNs are displayed in Table 4.

**Table 4:** ODNNs parameters.

Notation	Description
In	Input of neural networks
Out	Output of neural networks
*r*	Learning rate
*b*	Bias term
*w*	Weights
$\nabla_{w}$	Partial derivative with respect to weight
$\nabla_{b}$	Partial derivative with respect to bias

**(i). Initialization**

- Define the SFBHCR model’s parameters and initial conditions: θ, $\theta_{1}$, $\theta_{2}$, $\theta_{3}$, $\theta_{4}$, $\theta_{5}$, $\theta_{6}$, $\theta_{7}$, $\theta_{8}$, $\theta_{9}$, $\theta_{11}$, λ, μ.

**(ii). ODNNs Framework Setup**

- Input layer → **In**.
- Hidden Layers → **b, w, sine activation**
- Output Layer → **Out**

**(iii). Loss Function Formulation**

- Loss → **MSE**.

**(iv). Training Process**

- Generating the training data from SFBHCR model.
- Weights initialization → **w,b**.
- Optimization → Minimize Loss.
  - Update weights → **w** ← **w** – r.$\nabla_{w}$ Loss.
  - Update baises → **b** ← **b** – r.$\nabla_{b}$ Loss.

**(v). Prediction and evaluation**

- Validation → Use ODNNs for prediction.
  - Predicted outputs → **Out**.
  - Evaluation metrics → Accuracy.

**(vi). Hyperparameter Tuning**

- Adjusting hyperparameters → Layers, Neurons, Learning rate.
- Validation → Assess the model performance.

The steps for using ODNNs to simulate the SFBHCR model are shown in the above algorithm. The steps in the process are building the ODNNs, specifying the loss function to describe the SFBHCR dynamics, training the network, predicting outcomes, and modifying hyperparameters to enhance model performance.

### Numerical results and discussion

In this section, we will elaborate on the simulation outcomes attained through the utilization of the proposed method, ODNNs, and the traditional method, RK4. The values of the parameters given in Table 2. These simulations were conducted to examine the impacts of alcohol. Additionally, a comparison between both methods will be provided, along with a discussion on the advantageous aspects of ODNN, notably its adaptability to various step sizes.

### RK4 simulations

**Fig 5:**
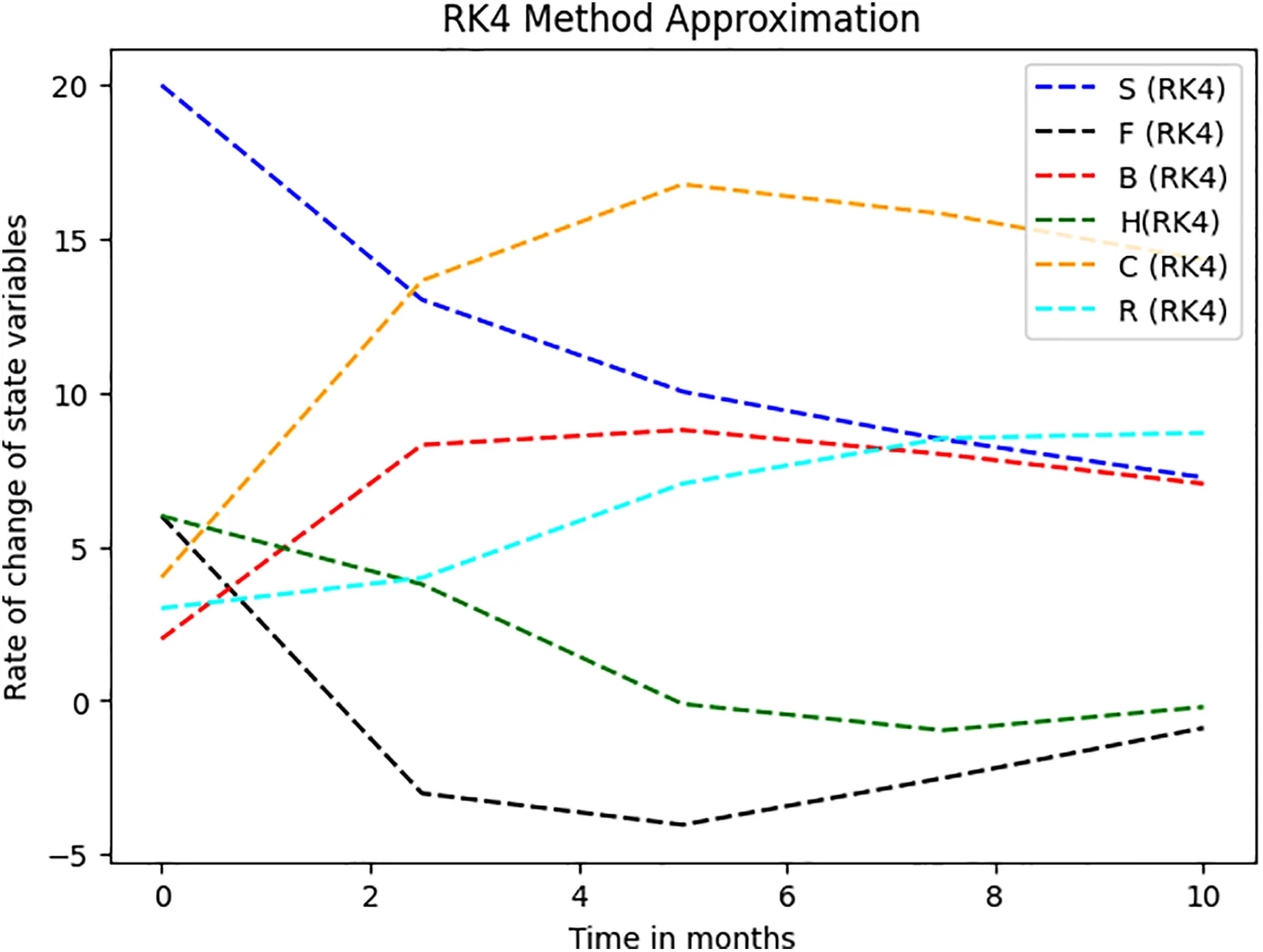
The profiles shows the physical interpretation of SFBHCR model obtained through RK4 method for *h* = 2.1.

**Fig 6:**
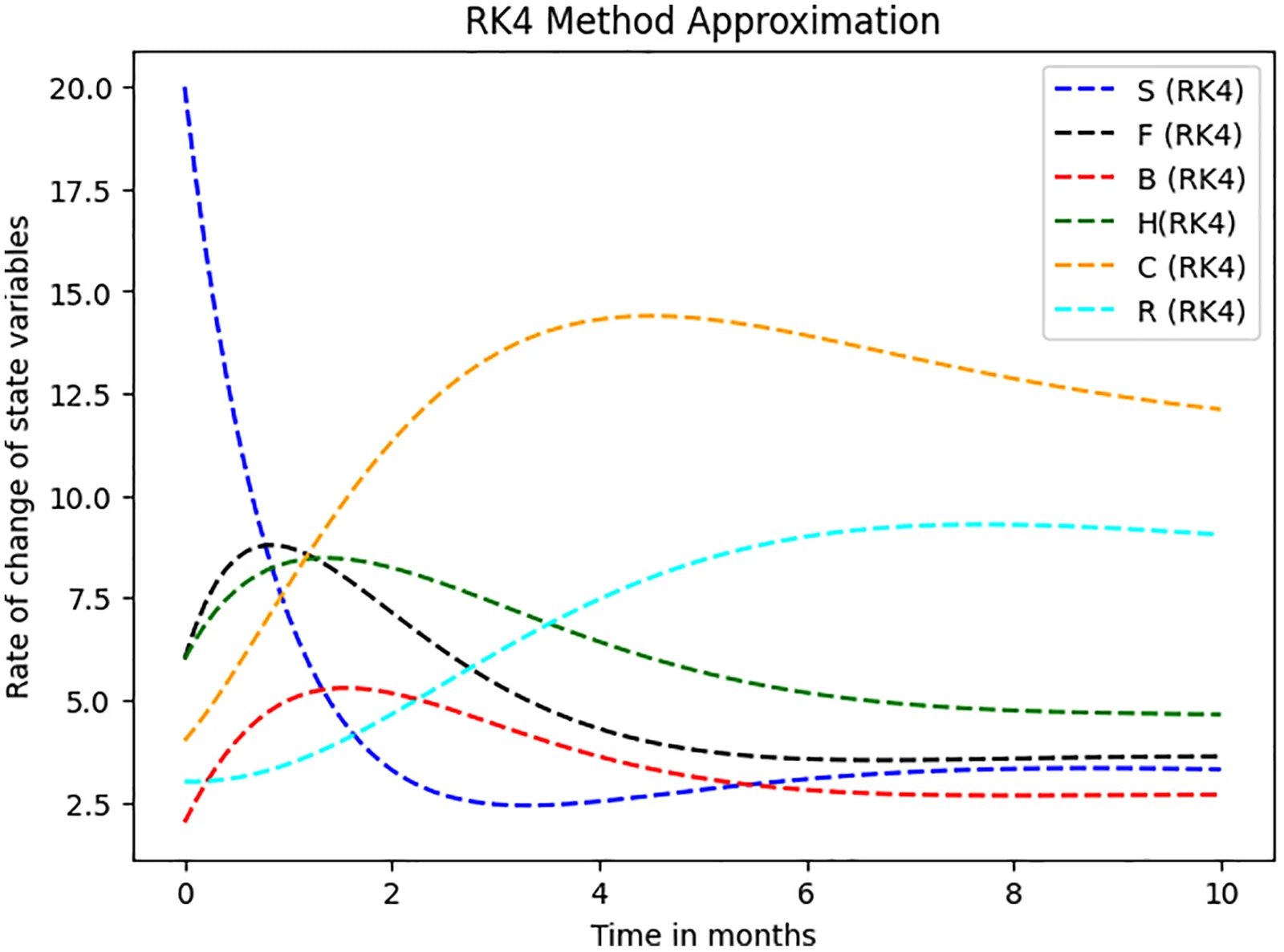
The profiles shows the physical interpretation of SFBHCR model obtained through RK4 method for *h* = 0.1.

Figs 5 and 6 illustrate the outcomes corresponding to two different step sizes, denoted as h. In Fig 5, for a step size of *h* = 2.1, the Runge-Kutta method of order four (RK4) yields negative values in compartment (F). Conversely, in Fig 6, RK4 yields positive results for smaller step sizes *h* = 0.1. It is notable that the efficacy of this method is contingent upon the step size and convergence criteria. Consequently, adopting larger step sizes can lead to undesirable outcomes, such as negativity and divergence, which are inherent drawbacks of the traditional RK4 approach.

### ODNNs simulations

The ODNNs method operates in a mesh-free manner, obviating the need for domain discretization and offering potential mitigation of certain limitations inherent in conventional numerical techniques such as RK4. Graphical representations validate that the proposed ODNNs approach effectively maintains convergence toward equilibrium points and upholds the essential positivity property.

**Fig 7:**
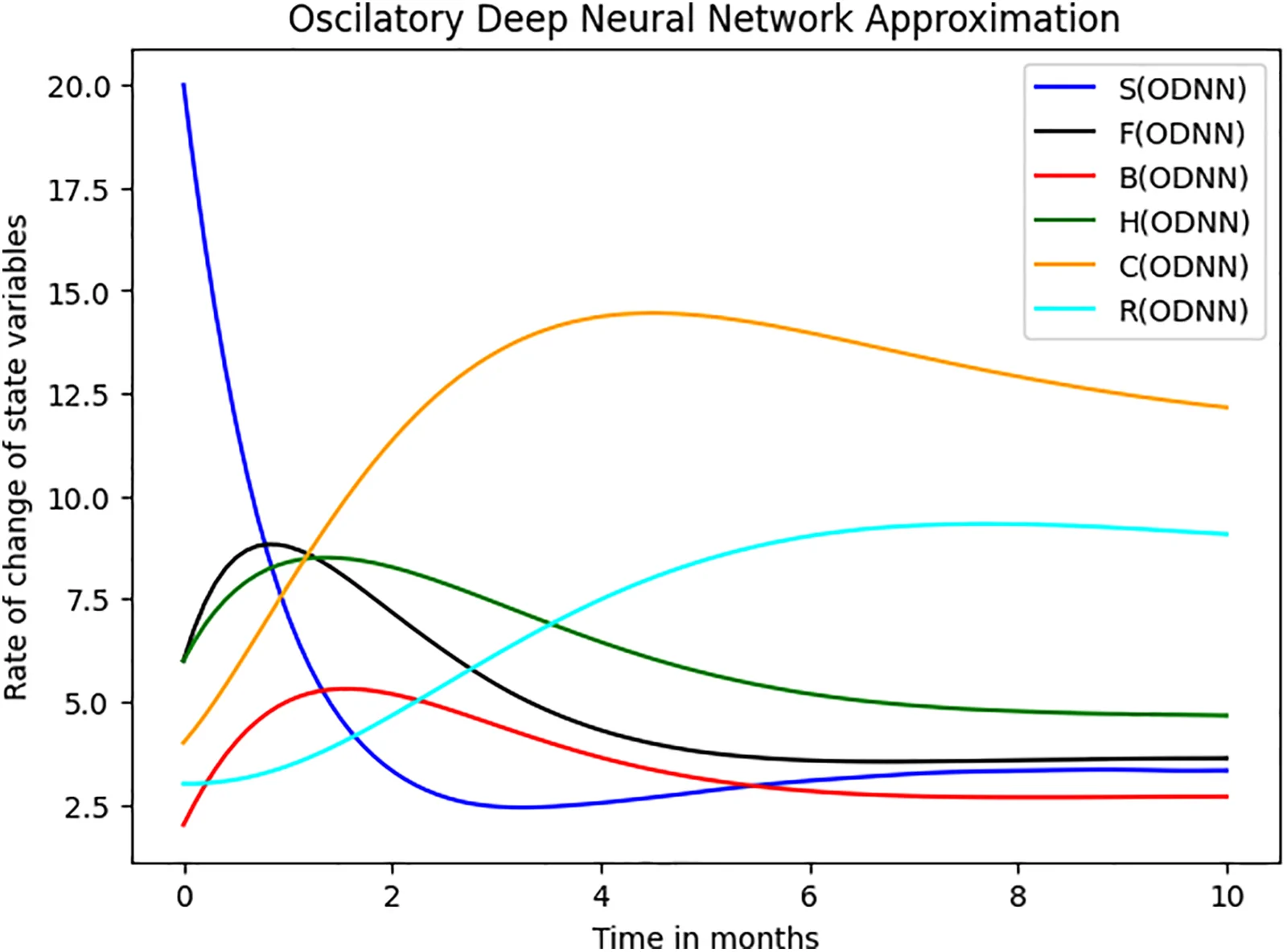
The profiles shows the dynamics of the nonlinear HCV model predicted by the proposed ODNNs architecture.

Fig 7 depicts the simulations of the SFBHCR model of HCV transmission under alcohol consumption, as predicted by the ODNNs method. Within a span of 35 days, 10 individuals from *S* enter the *F* compartment. Subsequently, after 145 days, 5 individuals who had commenced alcohol consumption reverted back to the susceptible compartment. Additionally, within 50 days, 8 individuals from *S* move to the *B* compartment. Following this, after 150 days, 5 individuals engaged in high alcohol consumption returned to the susceptible compartment. Over a period of 60 days, 5 susceptible people recover from the infection. Furthermore, within 90 days, 7 individuals initially engaged in lower levels of alcohol consumption escalated to high alcohol consumption, and after 170 days, individuals engaged in high alcohol consumption moved to lower levels. Moreover, within 40 days, 10 individuals from *H* progress to the *C* compartment. Finally, during a time frame of 80 days, 7 individuals who engaged in lower levels of alcohol consumption recovered from the infection. Within a span of 10 days, 5 individuals exhibiting high levels of alcohol consumption have successfully undergone recovery. Conversely, over the course of 35 days, ten individuals initially diagnosed with acute hepatitis C virus (HCV) have progressed to chronic stages, primarily due to the intricate nature of the disease. It is imperative to note that individuals moving to the chronic phase of HCV are at heightened risk of experiencing health-related complications post-recovery.

**Fig 8:**
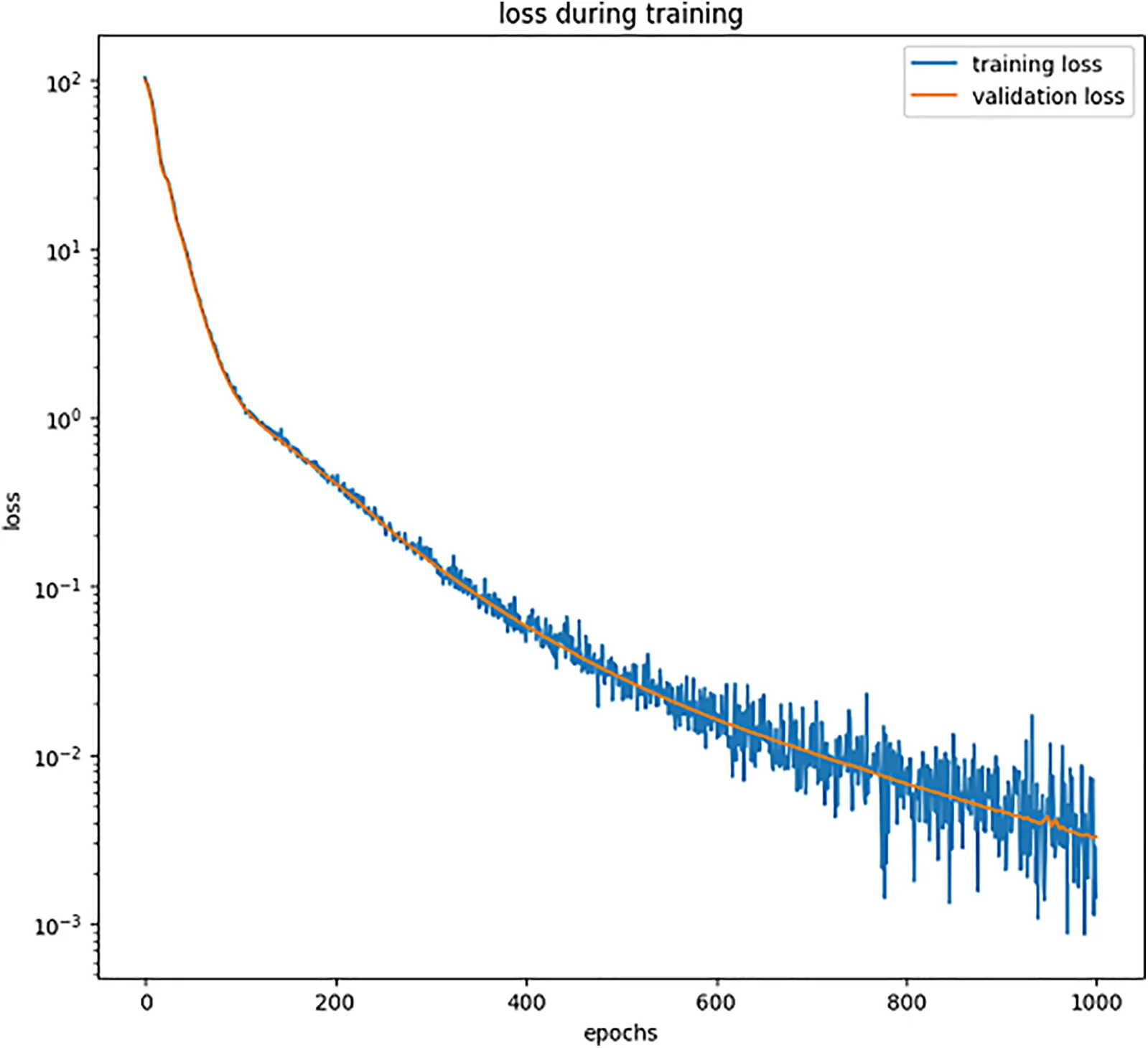
The graphs display the training and validation losses which represent the accuracy of the ODNNs.

In Fig 8, the training and validation loss are depicted. The training loss reflects the performance of the ODNNs on the training set by indicating the disparity between target values and predicted values throughout the training phase. Conversely, the validation loss signifies the performance of the ODNNs on unseen data, utilizing an independent dataset for computation. The trajectories of both training and validation losses illustrate the degree to which the ODNNs approximate solutions of the HCV transmission model. A decreasing trend in both training and validation losses over time suggests that the ODNNs effectively approximate the nonlinear models.

### Comparative analysis of simulations

**Fig 9:**
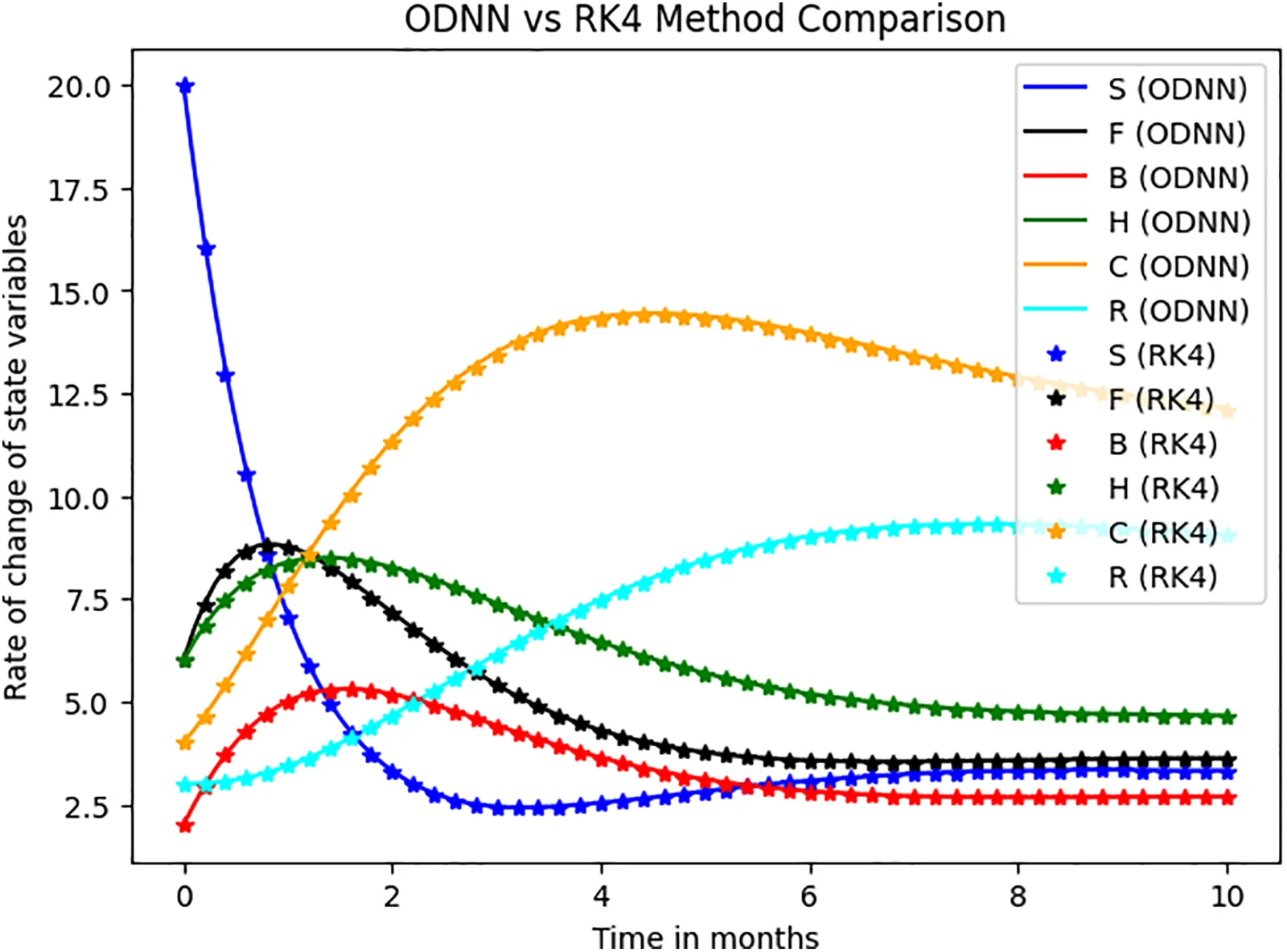
The profiles show the comparison between the ODNNs technique and RK4 method.

Fig 9 represents the comparison between the simulations obtained by RK4 and ODNNs. The static lines denote the RK4 approach, while the solid lines represent the ODNNs’ approximation. Utilizing RK4 and other numerical methodologies can entail significant computational costs, especially when applied to complex systems.

**Fig 10:**
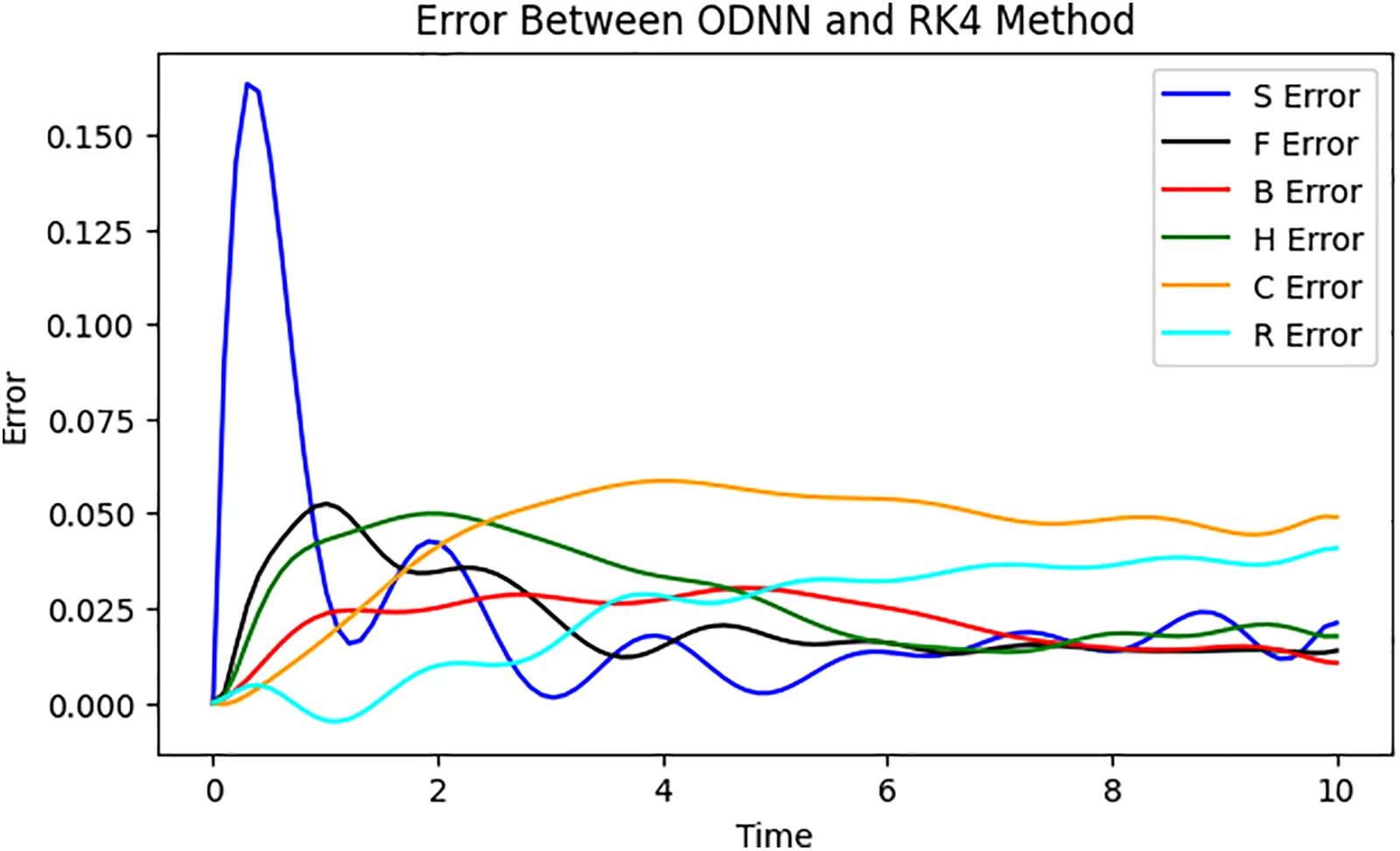
The profiles show the absolute error between the ODNNs technique and RK4 method.

To substantiate the proposed ODNNs-based approach, an error analysis was conducted, and the model’s accuracy was evaluated, as depicted in Fig 10. The graphical representation involves scrutinizing error fluctuations over time and across compartments. Comparatively, the predictions of the HCV model by ODNNs and RK4 exhibit closer alignment, manifesting in diminished error values. In Fig 10, it is discernible that ODNNs yield superior approximations, as evidenced by the convergence of error values across all compartments towards zero.

**Fig 11:**
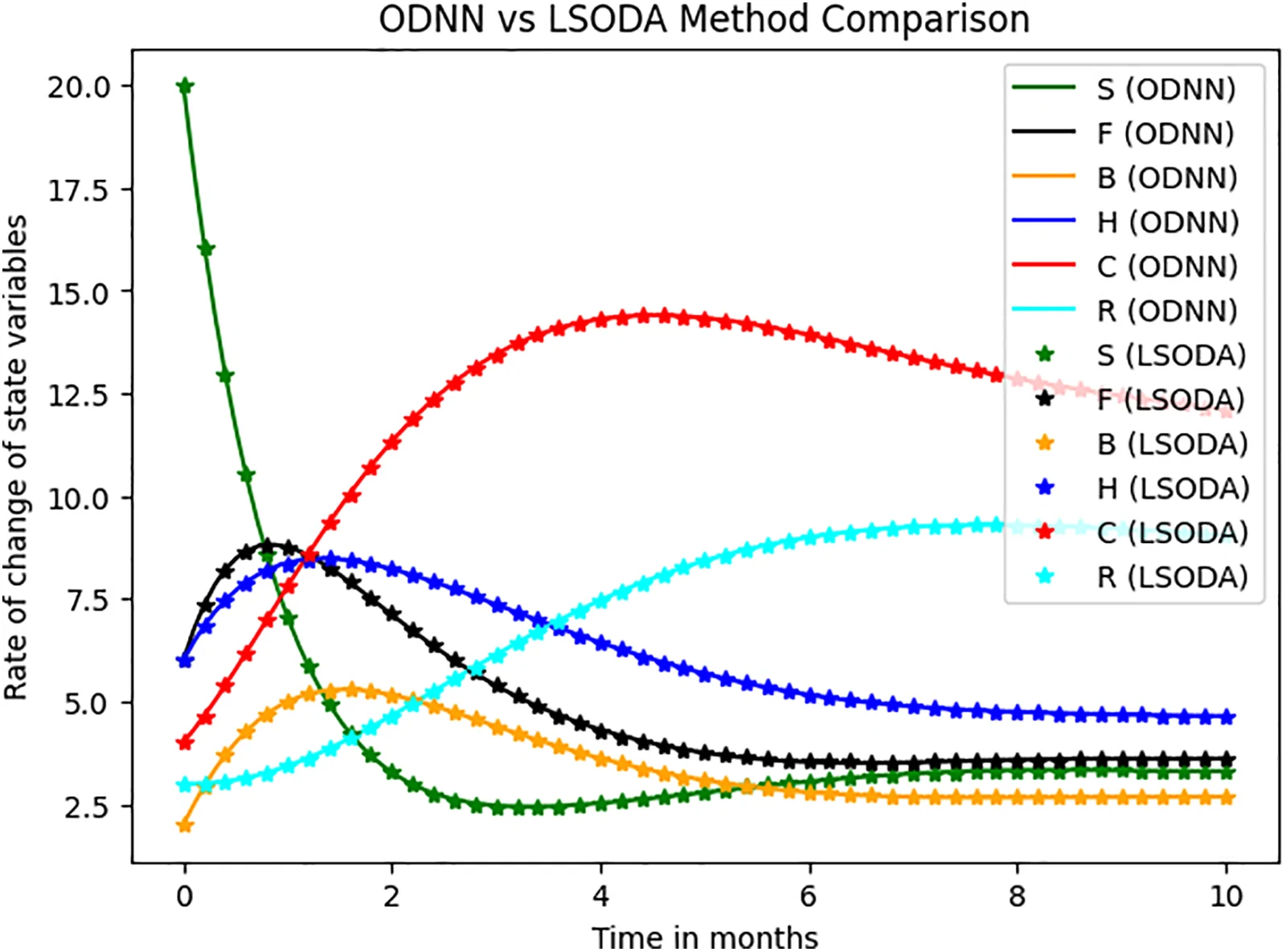
The profiles show the comparison between the ODNNs technique and LSODA method.

We present a comparative analysis of the results achieved using our proposed approach and those obtained through the LSODA algorithm, employing numerical methods such as Adams Bashforth and the Backward Differentiation Formula (BDF). Recently, the LSODA algorithm has been utilized to predict the transmission dynamics of the Marburg virus [54]. The comparison between the simulations obtained using LSODA and ODNNs is illustrated in Fig 11. The static lines represent the LSODA approach, while the solid lines depict the ODNNs’ approximation.

**Fig 12:**
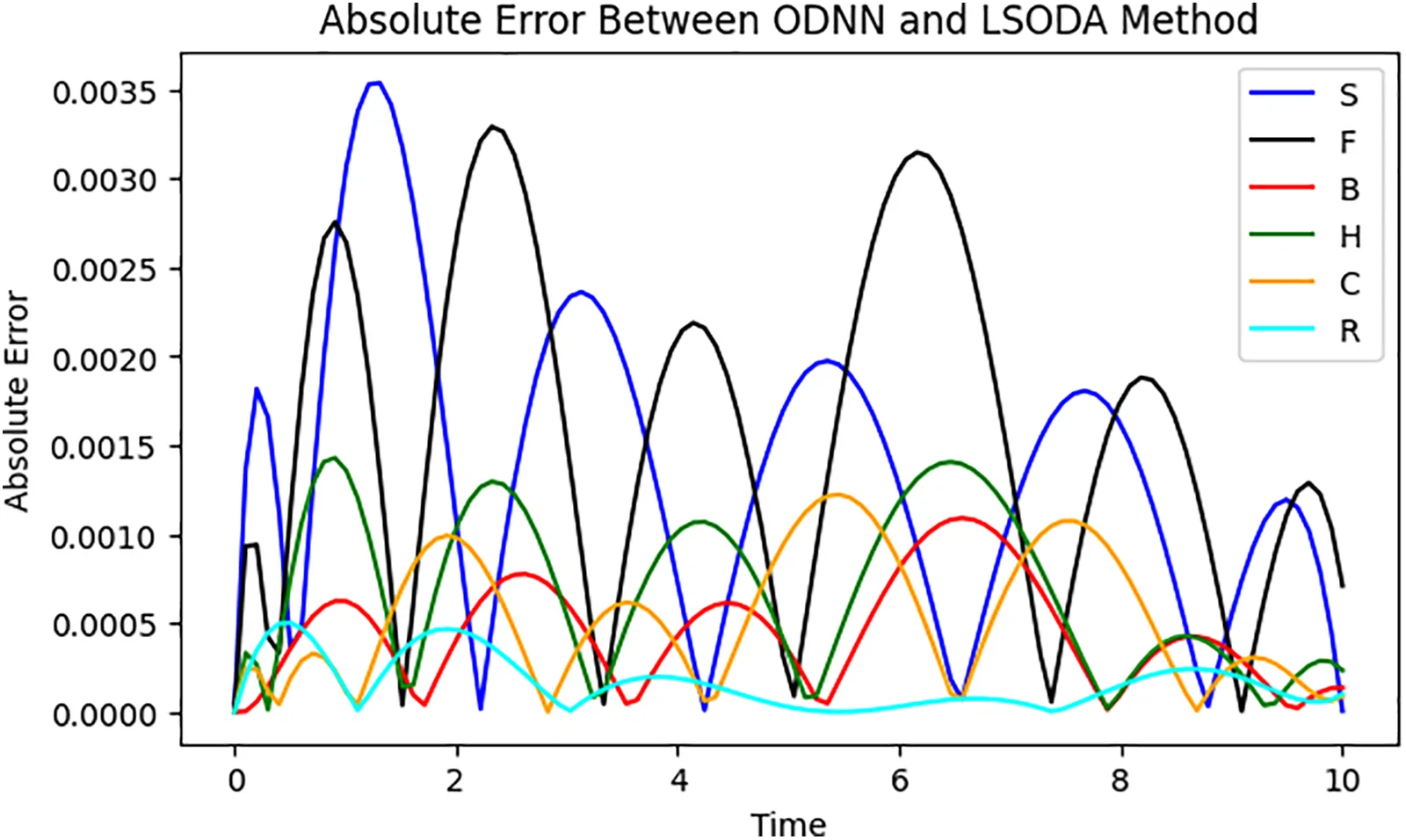
The profiles show the absolute error between the ODNNs technique and LSODA method.

To validate the proposed ODNNs-based approach an error analysis was performed and the model’s accuracy was assessed as shown in Fig 12.

From a practical and policy perspective, the proposed ODNN-based HCV model provides a useful computational framework for evaluating the impact of behavioral and medical interventions on disease transmission. The results demonstrate that alcohol-related behavioral transitions significantly influence the progression toward chronic infection, indicating that effective control strategies must address not only antiviral treatment but also alcohol consumption patterns. In particular, the model can assist public health authorities in assessing the potential effectiveness of intervention strategies such as early screening, awareness campaigns, alcohol reduction programs, and improved treatment adherence before their implementation. Moreover, the ability of the ODNN framework to generate stable and reliable predictions under different parameter settings makes it a valuable decision-support tool for planning and optimizing resource allocation in healthcare systems. Overall, the findings highlight that integrated intervention policies combining medical treatment with behavioral modification are essential for effective long-term control of hepatitis C transmission.

## Conclusion

In this paper, we developed a mathematical model of HCV transmission under the effect of boozing. The HCV model consists of six coupled nonlinear differential equations. For computational analysis of the nonlinear HCV model, we proposed an oscillatory deep neural network approach. The sine activation function is used in deep neural networks to investigate the dynamics of the model. The generated results are compared with the Runge-Kutta method and LSODA algorithm to verify the ODNNs’ accuracy. We simulated the nonlinear HCV transmission model using three different approaches and found that the classical Runge-Kutta does not converge to the equilibrium states of the model at each time step and does not preserve the positive characteristic. Even so, the suggested ODNNs approach maintains positivity and is unconditionally convergent.

The basic reproduction number *R*_0_ is calculated using the next-generation matrix method. The stability of the model is discussed at both the disease-free and endemic equilibrium points. The disease-free equilibrium point is locally and asymptotically stable. Additionally, a sensitivity analysis is performed to determine the most influential parameters in the model. Our extensive results show that the suggested approach works better than traditional approaches, such as the Runge-Kutta methods, and provides more reliability for epidemiological models. Along with methodological improvement, our research has clear policy implications for public health. The dominant contribution of alcohol-related factors to disease transmission indicates that the prevention of hepatitis C is not merely an issue of medical treatment but also of community-level interventions aimed at lowering alcohol consumption. Our computational method can be modified by policymakers to predict intervention policies and determine the most efficient deployment of resources, which makes this framework a useful tool for evidence-based decision-making.
